# Supplementary material for: Sedimentary ancient DNA reveals a threat of warming-induced alpine habitat loss to Tibetan Plateau plant diversity
Source: Nat Commun. 2021 May 20;12:2995. doi: 10.1038/s41467-021-22986-4 (PMC8137883; doi:10.1038/s41467-021-22986-4)
Supplement: Supplementary file 1 — Supplementary Information [file 41467_2021_22986_MOESM1_ESM.pdf]

Sedimentary ancient DNA reveals a threat of warming-induced alpine habitat loss to Tibetan Plateau plant diversity

Supplementary Information

Sisi Liu[1,2], Stefan Kruse[1], Dirk Scherler[3,4], Richard H. Ree[5], Heike H. Zimmermann[1], Kathleen R. Stoof-Leichsenring[1], Laura S. Epp[1], Steffen Mischke[6], and Ulrike Herzschuh[1,2,7*]

[1] *Alfred Wegener Institute Helmholtz Centre for Polar and Marine Research, Polar Terrestrial Environmental Systems, Potsdam, Germany*

[2] *Institute of Environmental Science and Geography, University of Potsdam, Potsdam, Germany*

[3] *GFZ German Research Centre for Geosciences, Potsdam, Germany*

[4] *Institute of Geological Sciences, Freie Universität Berlin, Berlin, Germany*

[5] *Negaunee Integrative Research Center, Department of Science and Education, Field Museum, Chicago, USA*

[6] *Institute of Earth Sciences, University of Iceland, Reykjavík, Iceland*

[7] *Institute of Biochemistry and Biology, University of Potsdam, Potsdam, Germany*

Supplementary discussion: Contamination in NTC6

After further sequence filtering (Methods), 32 unique sequence types (53,354 reads) were assigned to terrestrial seed plants in NTC6 in the batch with samples ESL011b-015b and ESL017b-019b. There was no positive gene band in the Agarose gel-electrophoresis, suggesting that the cross-contamination was unlikely to have occurred during the lab work. To check our supposition, we ran a principal component analysis (PCA) of all samples and NTC6. The PCA plot (Supplementary Figure 9) shows a wide difference between the sequence composition of NTC6 and the associated samples (batch 6, red dots), suggesting the origin of the contaminates in NTC 6 is unlikely to have come from this batch. Furthermore, there is no convincing evidence of cross-contamination among samples of batch 6 with batches 4 and 7 which were amplified with the same thermal cycler, as seen by their extremely different compositional taxa. In contrast, these samples were well repeated, as suggested by the similar taxa composition of replicates (e.g., ESL011a-015a and ESL017a-019a vs. ESL011b-015b and

ESL017b-019b, ESL021a-23a vs. ESL021b-23b and ESL028a vs. ESL028b). Taken together, we think that we used a wrong tag combination while pipetting the PCR mix for NTC6 but did not notice. This tag combination is unknown, and we cannot use it for demultiplexing. Because the actual sequence composition does not represent the true NTC for the corresponding PCR batch we did not remove those sequences detected in NTC6 from its controlled samples.

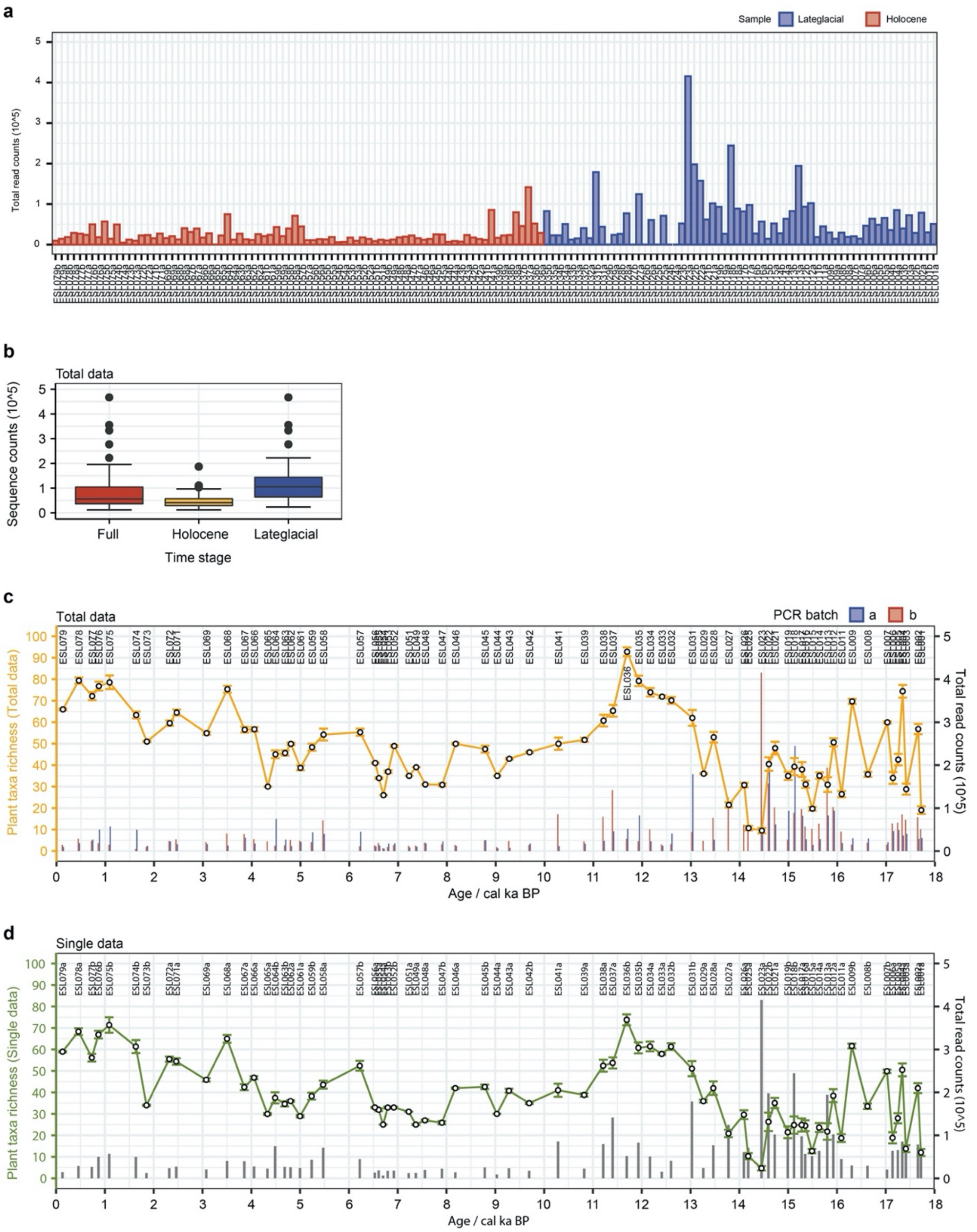

Supplementary Figure 1 Information about sedaDNA data with 100% best identity and plant taxa richness for sediments of Lake Naleng. a, The total read count of each PCR replicate of the lake-sediment samples. **b,** The box-and-whisker plots show that total read count per lake-sediment sample are comparable. Only a few outliers, marked as individual points, are present. The box plot represents the range of the middle 50% of the data, with a lower limit of 25th percentile, a median value, and an upper limit of 75th percentile. The set of whiskers indicate the date outside the middle 50%, excluding outliers. **c,** Plant taxa richness was calculated based on total data containing all positive PCR replicates (as colour bars indicate, n = 138 PCR replicates of 71 lake-sediment samples). **d,** Plant taxa richness was computed based on single data, consisting of one deeply sequenced PCR product that have a higher total read count within each sample (n = 71 PCR replicates of 71 lake-sediment samples). Before calculation, the sample ESL024 was excluded in both datasets due to no plant sedaDNA in both its PCR replicates. Data are presented as mean ± 95% confidence interval (error bars) in **c** and **d**. Source data are provided with this paper.

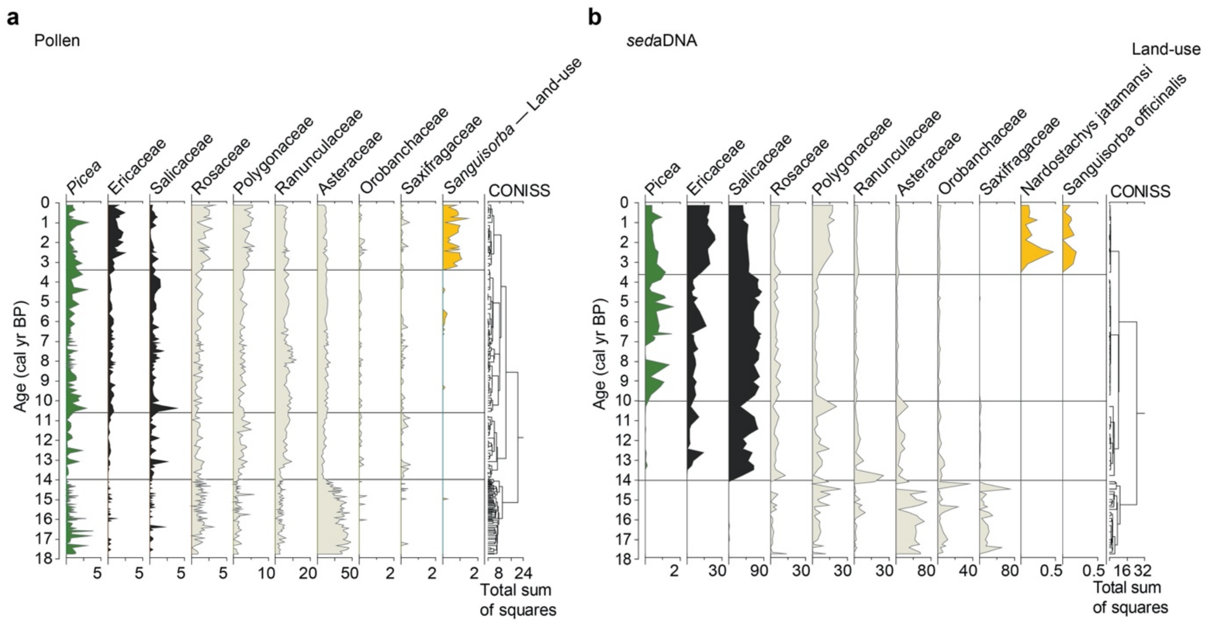

Supplementary Figure 2 Stratigraphic plots showing the relative (read) abundances of the dominant taxa and land-use indicators from the sediment core of Lake Naleng. A comparison between (a) pollen and (**b**) sedaDNA indicates that both proxies capture the major changes. However, differences suggest that the source of sedaDNA is restricted to Lake Naleng's catchment while pollen also comes from lower elevation sites. For example, Ericaceae and *Picea* are not detected before 14 ka, respectively in the sedaDNA record, consistent with the assumed climatic condition, while pollen of these taxa are present throughout the entire record.

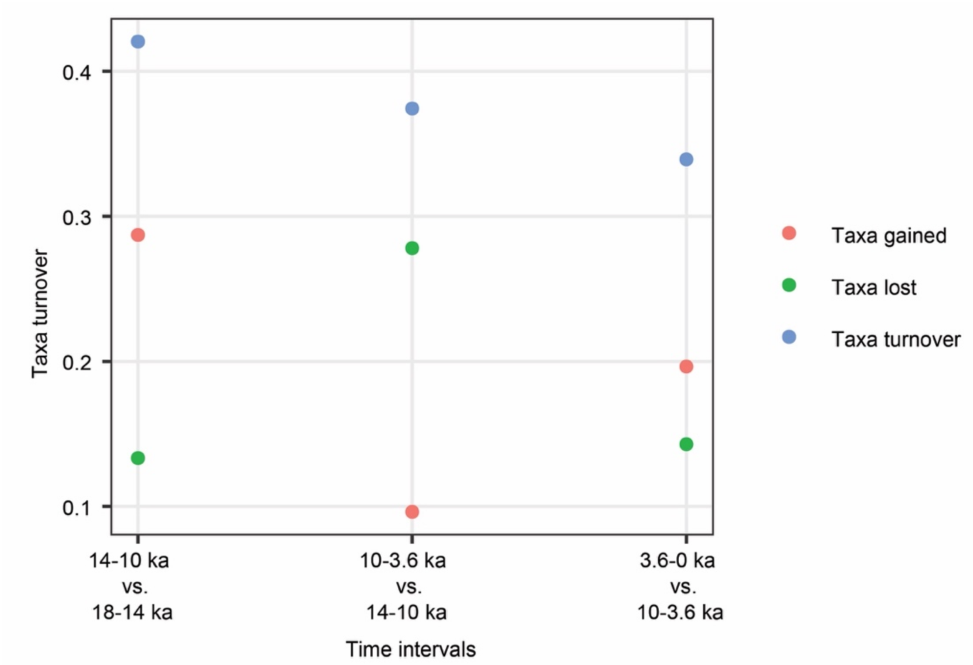

Supplementary Figure 3 Proportional plant taxa turnover since 18 ka. Taxa gain is defined as the proportion of immigrants that appear in the lake catchment between the selected time periods, while taxa lost is the proportional disappearance of species. Source data are provided in Supplementary Data 4.

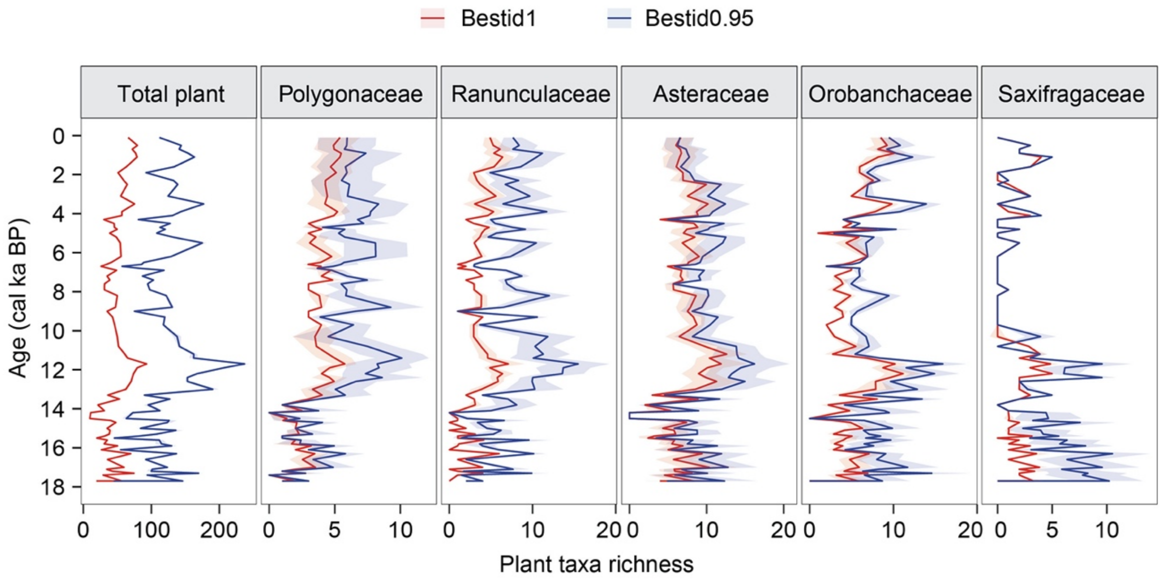

Supplementary Figure 4 Changes in plant taxa richness using different thresholds of best identity. Total plant taxa richness and within-family taxa richness based on sedaDNA data with 95% best identity (blue lines) compared to that of sedaDNA data with 100% best identity (red lines). Data are presented as mean ± 95% confidence interval (colour shading).

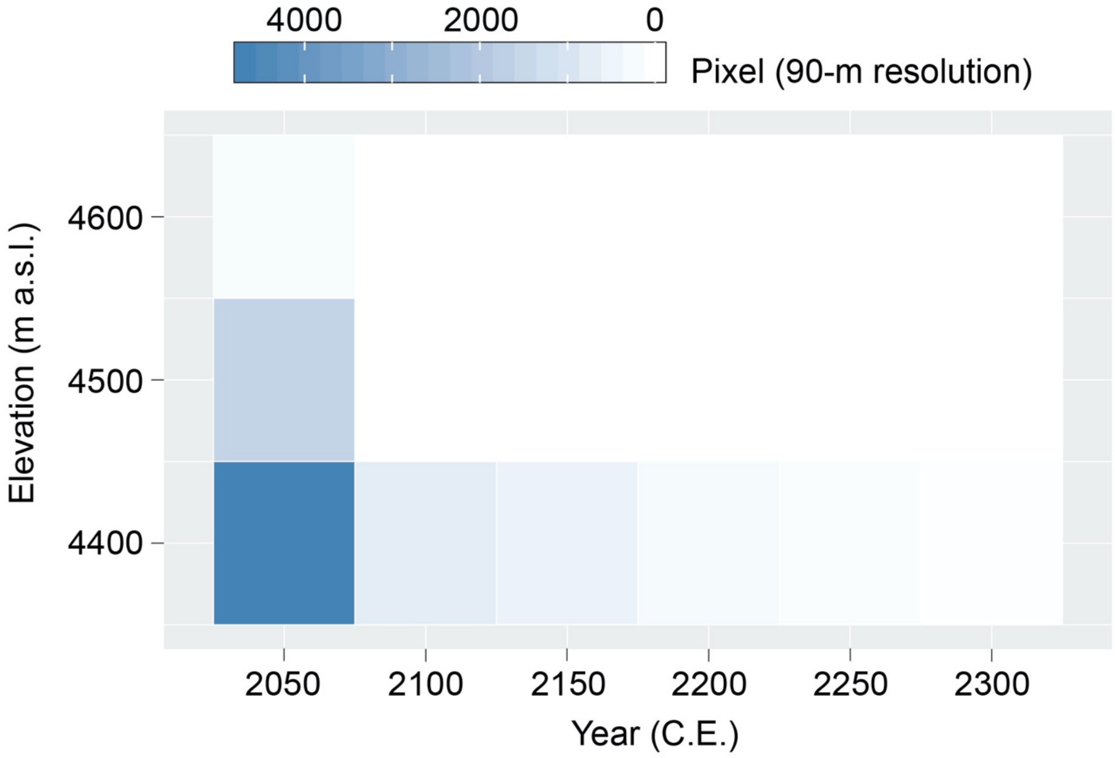

Supplementary Figure 5 Simulated alpine habitat loss under projected 2.5 °C warming future. Colour bar indicates the number of pixels (90-metre spatial resolution) within 100-m elevation bands above the treeline from 2050 C.E. to 2300 C.E. Source data are provided with this paper.

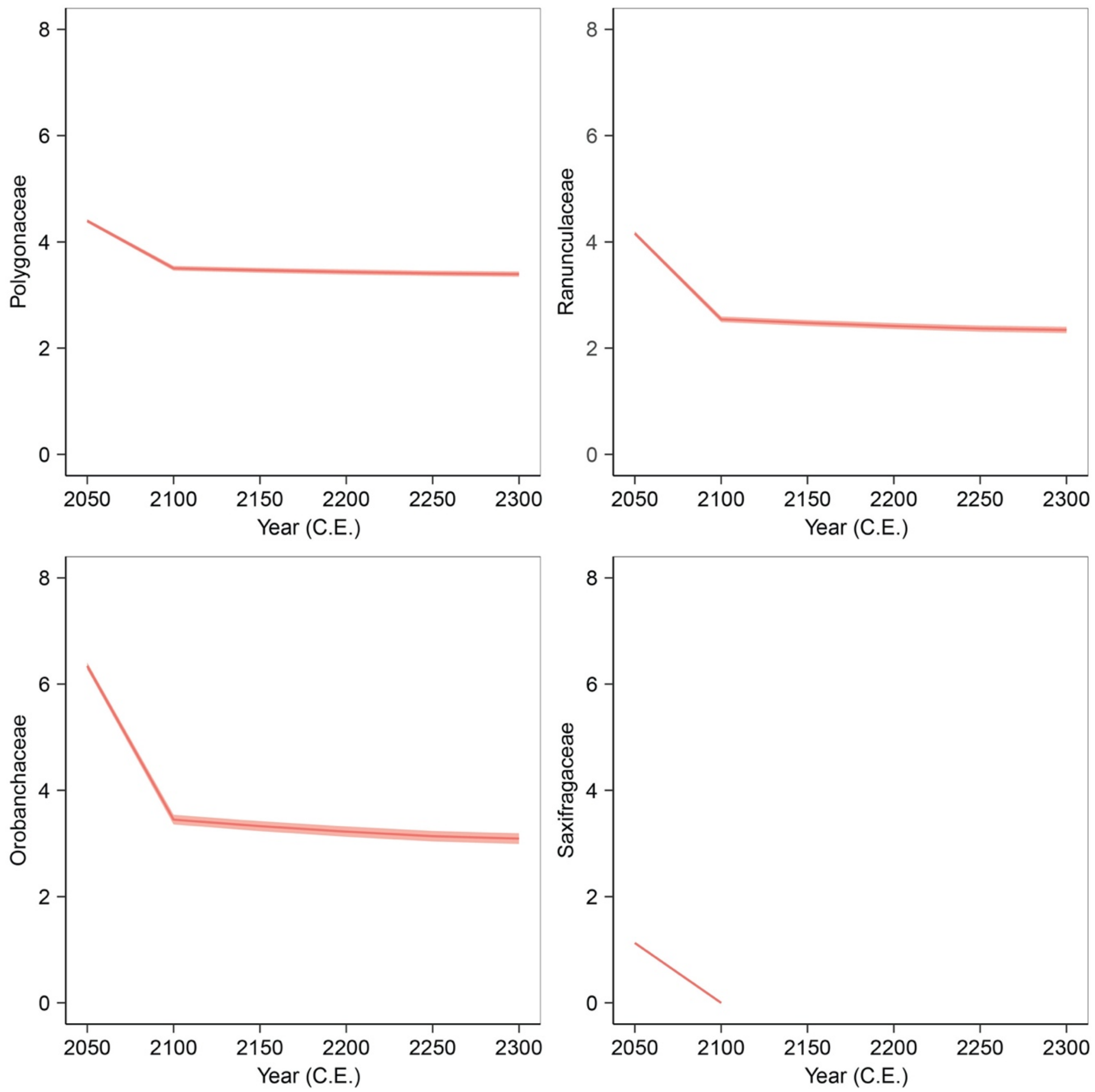

Supplementary Figure 6 Predicted loss of alpine plant taxa richness under the ongoing climate warming. Data are presented as mean ± 95% confidence interval (red shading).

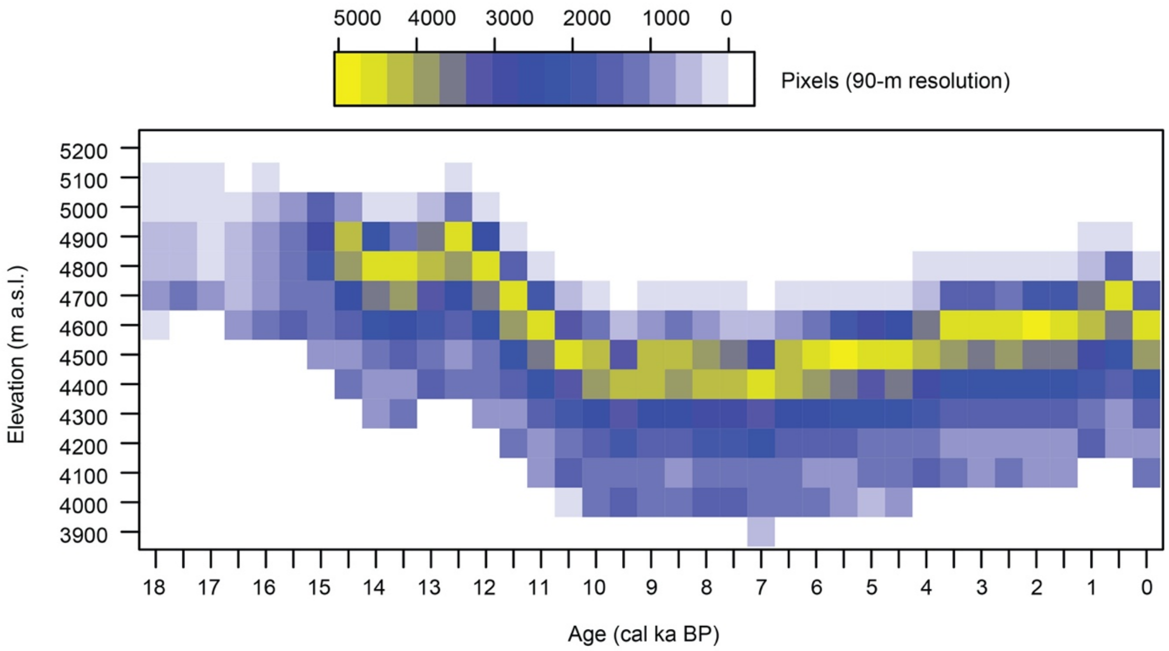

Supplementary Figure 7 Simulated area changes of available habitats represented by present elevations within Lake Naleng catchment since 18 ka. The available area for plant taxa in the catchment was predicted for 500-year steps based on the temperature lapse and reduced by potential glacier and permanent snow cover present at the beginning of the series. Current warming can be seen to drive changes towards warmer/lower elevations. Colour bar indicates the number of pixels (90-metre spatial resolution) within 100-m elevation bands across time. The pixels only shifted in elevational range from 3,900 to 5,200 m a.s.l. as indicated. Source data are provided with this paper.

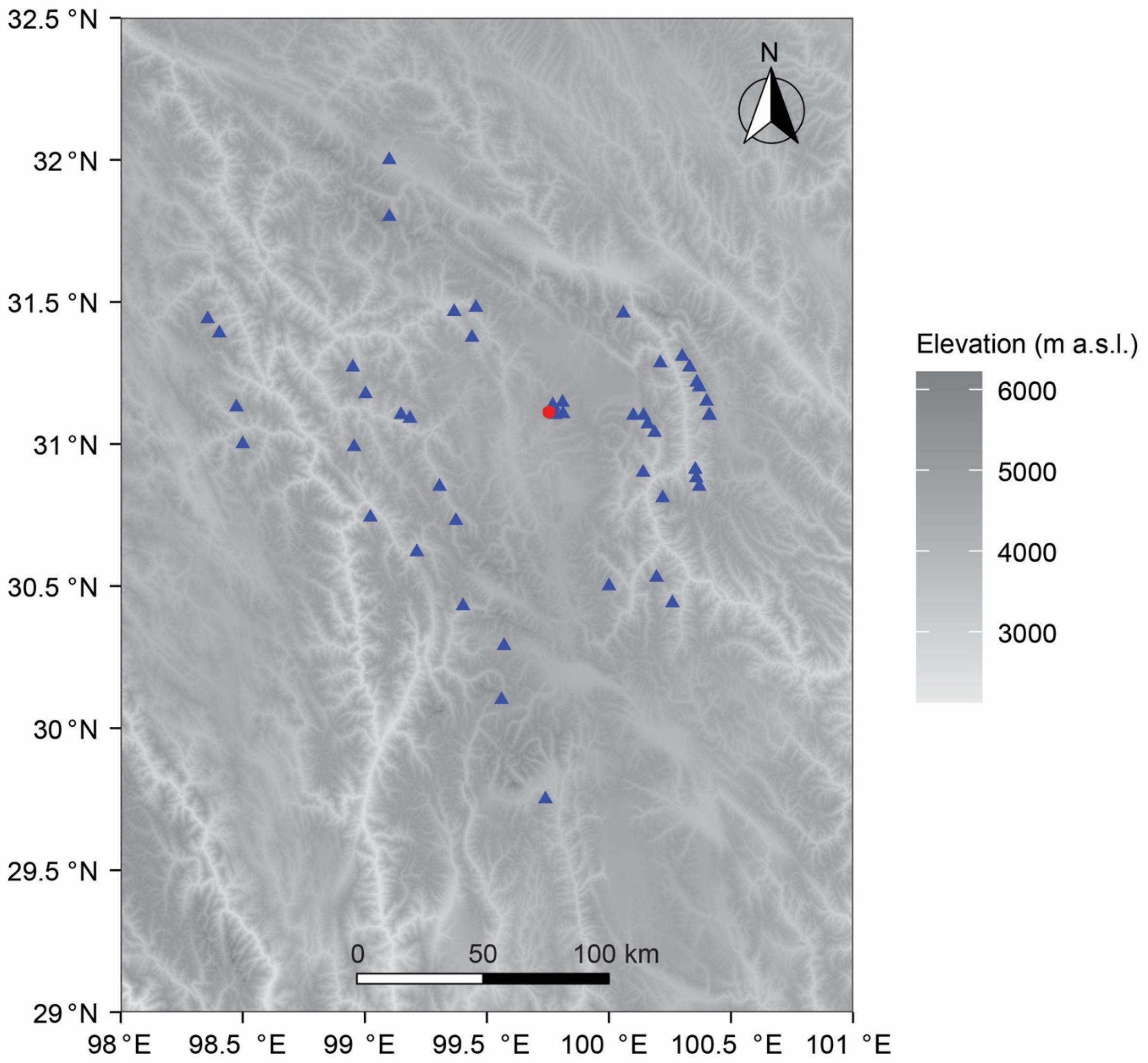

Supplementary Figure 8 The overview of treeline location (blue triangles) around Lake Naleng (red dot).

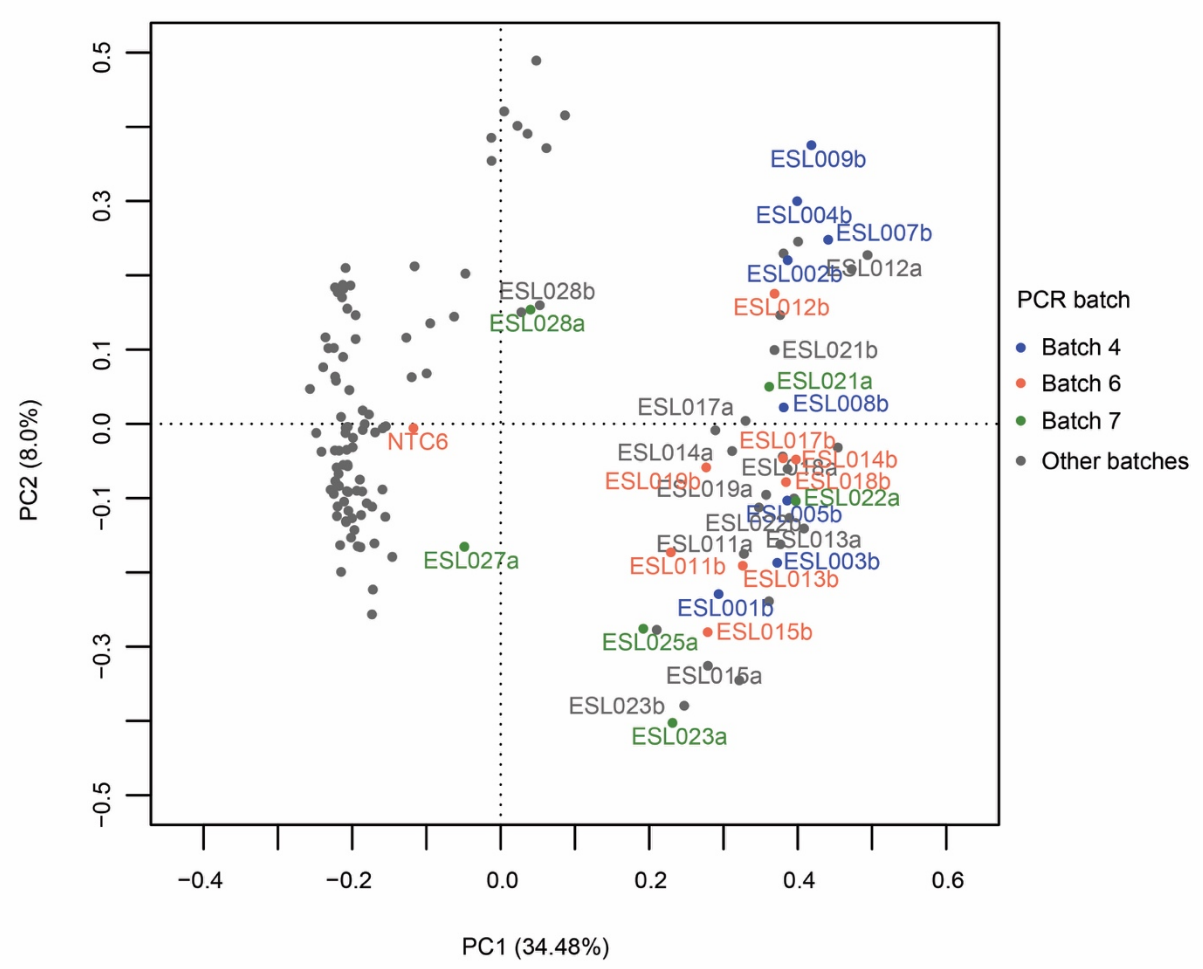

Supplementary Figure 9 The biplot of PCA results for all PCR replicates of lake sediment samples and NTC6. PCR Batches 4, 6 and 7 were amplified with same thermal cycler simultaneously.

Supplementary Table 1 The correlation of plant taxa richness based on different datasets indicates that the temporal variations of total plant taxa richness were accurately reflected by the data with best identity 1 in this study

	**rho**	**p-value**	**df**
**Bestid 1 vs. Non-ecotag data**	0.768	5.38e-15	69
**Bestid 1vs. terSeq data**	0.830	3.52e-19	69
**Bestid 1 vs. Bestid 0.95**	0.888	6.46e-25	69
**Bestid 1 vs. Single data**	0.951	7.74e-37	69

Bestid 1: after further sequence filtering, these are terrestrial seed plant sequences that have a best identity value of 1 and are present in at least two PCRs.
Bestid 0.95: after further sequence filtering, these are terrestrial seed plant sequences that have a best identity value ≥ 0.95 and are present in at least two PCRs.
terSeq: all terrestrial seed plant sequences without further sequence filtering.
Non-ecotag: data before taxonomic assignment
Single data: based on Bestid 1 data, but only using deeply sequenced PCR replicates for each lake-sediment sample.
rho: Spearman's Rank correlation coefficient
p-value: two tailed without adjustment.
df: degrees of freedom
adjusted: none
For detailed information, please see Methods.

Supplementary Table 2 Overview of Procrustes and Protest analyses

	p-value	r	m^{12}	rmse
**Bestid1 vs. Single data: samples**	0.001	0.986	0.027	0.020
**Bestid1 vs. Single data: taxa**	0.001	0.993	0.014	0.014

Bestid 1: after data quality control, these are terrestrial seed plant sequences that have a best identity value of 1 and are present in at least two PCRs.
Single data: based on Bestid 1 data, but only using deeply sequenced PCR replicates for each lake-sediment sample.
p-value: the smallest p-value with 999 permutations
r: Correlation in a symmetric Procrustes rotation
m^{12}: Procrustes sum of squares
rmse: Procrustes root mean squared error

Supplementary Table 3 Identified taxa and taxonomic resolution. We compared number of taxa and their taxonomic resolution of sedaDNA data with pollen data from the same sediment core. Taxonomic resolution is indicated by the percentage of taxa within each proxy.

	No. of Samples	No. of Taxa	Taxonomic resolution (%)						
			Subspecies	Species	Genus	Subtribe	Tribe	Subfamily	Family
**sedaDNA**	71	218	0.46	31.19	39.45	2.75	5.50	8.72	11.93
**Pollen**	191	152	0	23.03	63.82	0	0	1.32	11.84

Supplementary Table 4 Summary of correlation coefficients of taxa richness within the most common alpine families and selected predictor variables.

	18-10 ka					14-3.6 ka					10-0 ka				
	rho	adjusted *p*-value	df	adjusted df	alpha level	rho	adjusted *p*-value	df	adjusted df	alpha level	rho	adjusted *p*-value	df	adjusted df	alpha level
Polygonaceae															
Total habitat	**0.693**	**1.13e-05**	**34**	**10**	**.025**			/					/		
Temperature	**0.673**	**2.77e05**	**34**	**11**	**.025**	-0.276	0.394	35	13	.25	**-0.882**	**1.05e-11**	**33**	**12**	**.0005**
Glacier's decay	**-0.634**	**3.86e-04**	**30**	**13**	**.025**			/					/		
Alpine habitat			/			0.277	0.386	35	13	.25	**0.908**	**1.92e-13**	**33**	**11**	**.0005**
Mg/Ca ratio	**0.706**	**6.17e-06**	**34**	**11**	**.025**	0.190	1.000	35	12	>.25	0.117	1.000	33	11	>.25
Land-use			/					/			**0.937**	**4.83e-16**	**33**	**11**	**.0005**
Ranunculaceae															
Total habitat	**0.605**	**3.67e-04**	**34**	**10**	**.05**			/					/		
Temperature	**0.582**	**7.93e-04**	**34**	**11**	**.05**	**-0.731**	**1.13e-07**	**35**	**11**	**.01**	**-0.954**	**2.79e-18**	**33**	**11**	**.0005**
Glacier's decay	-0.265	0.573	30	13	.25			/					/		
Alpine habitat			/			**0.739**	**7.14e-07**	**35**	**11**	**.01**	**0.982**	**5.78e-25**	**33**	**11**	**.0005**
Mg/Ca ratio	**0.688**	**1.43e-05**	**34**	**11**	**.025**	0.384	0.075	35	11	.25	0.184	1.000	33	11	>.25
Land-use			/					/			**0.935**	**7.99e-16**	**33**	**11**	**.0005**
Asteraceae															
Total habitat	0.283	0.379	34	10	.25			/					/		
Temperature	0.261	0.498	34	11	.25	-0.428	0.33	35	11	.10	-0.032	1.000	33	13	>.25
Glacier's decay	**-0.704**	**2.76e-05**	**30**	**13**	**.005**			/					/		
Alpine habitat			/			0.431	0.031	35	11	.10	0.058	1.000	33	12	>.25
Mg/Ca ratio	0.354	0.137	34	11	.25	0.452	0.020	35	11	.10	0.325	0.227	33	13	.25
Land-use			/					/			-0.064	1.000	33	13	>.25
Orobanchaceae															
Total habitat	**0.572**	**0.001**	**34**	**10**	**.05**			/					/		
Temperature	0.520	0.005	34	11	.10	**-0.871**	**9.15e-12**	**35**	**11**	**.001**	**-0.896**	**1.32e-12**	**33**	**11**	**.0005**
Glacier's decay	**-0.699**	**3.47e-05**	**30**	**12**	**.01**			/					/		
Alpine habitat			/			**0.879**	**3.41e-12**	**35**	**11**	**.0005**	**0.918**	**3.67e-14**	**33**	**11**	**.0005**
Mg/Ca ratio	**0.585**	**0.001**	**34**	**10**	**.05**	0.339	0.161	35	10	.25	0.007	1.000	33	11	>.25
Land-use			/					/			**0.969**	**6.23e-21**	**33**	**11**	**.0005**
Saxifragaceae															
Total habitat	0.002	1.000	34	11	>.25			/					/		
Temperature	-0.031	1.000	34	11	>.25	**-0.889**	**7.70e-13**	**35**	**11**	**.0005**	**-0.915**	**5.75e-14**	**33**	**11**	**.0005**
Glacier's decay	**-0.496**	**0.016**	**30**	**13**	**.05**			/					/		
Alpine habitat			/			**0.900**	**1.48e-13**	**35**	**11**	**.0005**	**0.939**	**3.23e-16**	**33**	**10**	**.0005**
Mg/Ca ratio	0.154	1.000	34	11	>.25	**0.496**	**0.007**	**35**	**10**	**.10**	0.113	1.000	33	11	>.25
Land-use			/					/			**0.949**	**1.73e-17**	**33**	**11**	**.0005**

rho: Spearman's Rank correlation coefficient
adjusted *p*-value: two tailed with Bonferroni adjustment
df: degrees of freedom
adjusted df: adjusted degrees of freedom
alpha level: Directional alpha levels of critical values for Spearman's Rank correlation coefficient
/: not a predictor variable in corresponding time transition
predictor variable with alpha level ≤ 0.05 was in bold.

Supplementary Table 5 Summary of generalised linear model (GLM) diagnostics for total plant taxa richness and taxa richness within the most common alpine families during 10–0 ka.

Family	Model	Formula	AIC	Deviance explained of model (%)	P-value and correlation	varImp(): variable importance
Total richness	Null model	sRichness ~ 1, family = gaussian,	277.73	0	NA	NA
	model 1	sRichness ~ Aa, family = gaussian,	166.74	96.04	1.03E-24 ***/+	NA
	model 2	sRichness ~ sHuman, family = gaussian,	210.14	86.31	8.25E-16 ***/+	NA
	model 3	sRichness ~ Aa + sHuman, family = gaussian,	163.98	96.54	Aa: 4.39E-11 ***/+	Aa: 9.73
					sHuman: 0.038 */+	sHuman: 2.16
	model 4	backwards = step(model3)	163.98	96.54	sHuman: 0.038 */+	sHuman: 2.16
					Aa: 4.39E-11 ***/+	Aa: 9.73
Orobanchaceae	Null model	sRichness ~ 1, family = gaussian,	140.62	0	NA	NA
	model 1	sRichness ~ Aa, family = gaussian,	27.59	96.26	3.95E-25 ***/+	NA
	model 2	sRichness ~ sHuman, family = gaussian,	75.21	85.43	2.35E-15 ***/+	NA
	model 3	sRichness ~ Aa + sHuman, family = gaussian,	26.50	96.58	Aa: 1.35E-11 ***/+	Aa: 10.21
					sHuman: 0.095 /+	sHuman: 1.72
	model 4	backwards = step(model3)	26.50	96.58	sHuman: 0.095 /+	sHuman: 1.72
					Aa: 1.35E-11 ***/+	Aa: 10.21
Polygonaceae	Null model	sRichness ~ 1, family = gaussian,	60.29	0	NA	NA
	model 1	sRichness ~ Aa, family = gaussian,	-21.51	90.88	1.01E-18 ***/+	NA
	model 2	sRichness ~ sHuman, family = gaussian,	-51.17	96.09	8.28E-25 ***/+	NA
	model 3	sRichness ~ Aa + sHuman, family = gaussian,	-69.87	97.84	Aa: 1.57E-05 ***/+	Aa: 5.08
					sHuman: 1.59E-11 ***/+	sHuman: 10.14
	model 4	backwards = step(model3)	-69.87	97.84	sHuman: 1.59E-11 ***/+	sHuman:10.14
					Aa: 1.57E-05 ***/+	Aa: 5.08
Ranunculaceae	Null model	sRichness ~ 1, family = gaussian,	100.10	0	NA	NA
	model 1	sRichness ~ Aa, family = gaussian,	-8.76	95.79	2.82E-24 ***/+	NA
	model 2	sRichness ~ sHuman, family = gaussian,	45.45	80.19	3.84E-13 ***/+	NA
	model 3	sRichness ~ Aa + sHuman, family = gaussian,	-6.87	95.80	Aa: 2.58E-12 ***/+	Aa: 10.91
					sHuman: 0.755/+	sHuman: 0.31
	model 4	backwards = step(model3)	-6.87	95.80	sHuman: 0.755/+	sHuman: 0.31
					Aa: 2.58E-12 ***/+	Aa: 10.91
Saxifragaceae	Null model	sRichness ~ 1, family = gaussian,	76.40	0	NA	NA
	model 1	sRichness ~ Aa, family = gaussian,	-21.94	94.31	4.04E-22 ***/+	NA
	model 2	sRichness ~ sHuman, family = gaussian,	28.07	76.26	7.75E-12 ***/+	NA
	model 3	sRichness ~ Aa + sHuman, family = gaussian,	-21.53	94.57	Aa:9.00E-12 ***/+	Aa: 10.38
					sHuman: 0.2326/+	sHuman: 1.22
	model 4	backwards = step(model3)	-21.53	94.57	sHuman: 0.2326/+	sHuman: 1.22
					Aa: 9.00E-12 ***/+	Aa: 10.38

sRichness: smoothed data of plant taxa richness
sHuman: smoothed data of grazing indicator
Aa: alpine habitat area
Significance codes: *** 0.001, ** 0.01, * 0.05
Correlation code: all correlations are positive (+)
NA: no data/without calculation

Supplementary Table 6 List of primers used for plant sedaDNA amplification

**DNA region**	**Name**	**Sequence 5'-3'**	**Amplicon length range (bp)**
trnL (UAA) P6 loop	g	GGGCAATCCTGAGCCAA	10-143 bp
	h	CCATTGAGTCTCTGCACCTATC	

Supplementary Table 7 The geographic information of treeline location around Lake Naleng

ID	Latitude / °N	Longitude/ °E	Elevation/ m a.s.l.	Source
Shaluli Shan S	29.75	99.74	4200	Publication[1]
NE of Batang	30.1	99.56	4500	Publication[1]
Haizi Shan	30.29	99.57	4200	Publication[1]
Chola Shan	32	99.1	4200	Publication[2]
NW of Litang	30.5	100	4650	Publication[3]
SE of Zogqen	31.8	99.1	4350	Publication[3]
Gonjo-Jundah	31	98.5	3900	Publication[4]
1	31.13	99.77	4365	Google Earth
2	31.13425	99.77	4404	Google Earth
3	31.137	99.77	4355	Google Earth
4	31.146	99.81	4424	Google Earth
5	31.103	99.78	4398	Google Earth
6	31.106	99.812	4435	Google Earth
7	31.46	100.059	4335	Google Earth
8	31.285	100.21	4396	Google Earth
9	31.308	100.3	4520	Google Earth
10	31.27	100.33	4502	Google Earth
11	31.217	100.36	4512	Google Earth
12	31.2	100.37	4362	Google Earth
13	31.15	100.4	4342	Google Earth
14	31.1	100.41	4341	Google Earth
15	31.1	100.413	4377	Google Earth
16	30.91	100.354	4203	Google Earth
17	30.88	100.358	4396	Google Earth
18	30.85	100.37	4222	Google Earth
19	31.1	100.1	4125	Google Earth
20	31.1	100.142	4521	Google Earth
21	31.07	100.159	4323	Google Earth
22	31.04	100.186875	4386	Google Earth
23	30.9	100.14	4267	Google Earth
24	30.81	100.22	4493	Google Earth
25	30.53	100.195	4388	Google Earth
26	30.44	100.26	4516	Google Earth
27	30.43	99.402	4434	Google Earth
28	30.62	99.213	4445	Google Earth
29	30.73	99.373	4280	Google Earth
30	30.85	99.306	4427	Google Earth
31	30.742	99.0229	4250	Google Earth
32	30.99	98.956	4245	Google Earth
33	31.09	99.185	4462	Google Earth
34	31.103	99.148	4402	Google Earth
35	31.176	99.003	4264	Google Earth
36	31.27	98.9507	4194	Google Earth
37	31.4657	99.366	4102	Google Earth
38	31.48	99.455	4305	Google Earth
39	31.3749	99.4387	4265	Google Earth
40	31.13	98.474	4329	Google Earth
41	31.39	98.404	4429	Google Earth
42	31.44	98.3562	4526	Google Earth

References Supplementary Table 7:

1. Yao, Y. & Zhang, B. The mass elevation effect of the Tibetan Plateau and its implications for Alpine treelines. *Int. J. Climatol* **35**, 1833–1846 (2015).
2. Jiang, F., Wu, X., Wang, S., Fu, J., Wang, Y. Features of space distribution of the forest line and relations between the forest line and climatic limit of permafrost and climatic snowline in China (in Chinese). *J. Geomech*. **10**, 289–299 (2004).
3. Schickhoff, U. The Upper Timberline in the Himalayas, Hindu Kush and Karakorum: a Review of Geographical and Ecological Aspects. in *Mountain Ecosystems* (eds. Broll, G. & Keplin, B.) 275–354 (Springer-Verlag, 2005).
4. Zhang, J.W., Chen, W.J., Li, W., Zhao, B.K. *The Vegetation of Xizang* (in Chinese). (Science Press: Beijing, 1988)
