# Supplementary material for: Sedimentary ancient DNA reveals a threat of warming-induced alpine habitat loss to Tibetan Plateau plant diversity
Source: Nat Commun. 2021 May 20;12:2995. doi: 10.1038/s41467-021-22986-4 (PMC8137883; doi:10.1038/s41467-021-22986-4)
Supplement: Supplementary file 3 — Description of Additional Supplementary Files [file 41467_2021_22986_MOESM3_ESM.pdf]

Description of Additional Supplementary Files

File Name: Supplementary Data 1
Description: The bestid 1 dataset

File Name: Supplementary Data 2
Description: The bestid 0.95 dataset

File Name: Supplementary Data 3
Description: Data for statistical analysis

File Name: Supplementary Code 1
Description: Rarefaction

File Name: Supplementary Code 2
Description: Taxa turnover

File Name: Supplementary Code 3
Description: Multiple correlation

File Name: Supplementary Code 4
Description: Adjust df

File Name: Supplementary Code 5
Description: GLMs and prediction
