# Supplementary material for: Sedimentary ancient DNA reveals a threat of warming-induced alpine habitat loss to Tibetan Plateau plant diversity
Source: Nat Commun. 2021 May 20;12:2995. doi: 10.1038/s41467-021-22986-4 (PMC8137883; doi:10.1038/s41467-021-22986-4)
Supplement: Supplementary file 6 — Reporting Summary [file 41467_2021_22986_MOESM6_ESM.pdf]

nature research

Corresponding author(s): Ulrike Herzschuh

Last updated by author(s): Mar 15, 2021

Reporting Summary

Nature Research wishes to improve the reproducibility of the work that we publish. This form provides structure for consistency and transparency in reporting. For further information on Nature Research policies, see Authors & Referees and the Editorial Policy Checklist .

Statistics

For all statistical analyses, confirm that the following items are present in the figure legend, table legend, main text, or Methods section.

n/a	Confirmed	
☐	☒	The exact sample size (n) for each experimental group/condition, given as a discrete number and unit of measurement
☐	☒	A statement on whether measurements were taken from distinct samples or whether the same sample was measured repeatedly
☐	☒	The statistical test(s) used AND whether they are one- or two-sided<br>*Only common tests should be described solely by name; describe more complex techniques in the Methods section.*
☐	☒	A description of all covariates tested
☐	☒	A description of any assumptions or corrections, such as tests of normality and adjustment for multiple comparisons
☐	☒	A full description of the statistical parameters including central tendency (e.g. means) or other basic estimates (e.g. regression coefficient) AND variation (e.g. standard deviation) or associated estimates of uncertainty (e.g. confidence intervals)
☐	☒	For null hypothesis testing, the test statistic (e.g. F, t, r) with confidence intervals, effect sizes, degrees of freedom and P value noted<br>*Give P values as exact values whenever suitable.*
☒	☐	For Bayesian analysis, information on the choice of priors and Markov chain Monte Carlo settings
☒	☐	For hierarchical and complex designs, identification of the appropriate level for tests and full reporting of outcomes
☐	☒	Estimates of effect sizes (e.g. Cohen's d, Pearson's r), indicating how they were calculated

Our web collection on statistics for biologists contains articles on many of the points above.

Software and code

Policy information about availability of computer code

Data collection	Google Earth was used to collect the elevation of treeline (see Supplementary Table 7).
Data analysis	OBITools package (version 1.2.13) for DNA sequencing data analysis that deposited on Python 2.7, which can be install in UNIX-like operating system. The ObiTools code is available on Dryad digital repository with identifier doi:10.5061/dryad.vdncjsxth. The glacial modeling is initially implemented in MATLAB (version 8.6). But the script can be run in GNU Octave (open access, version 4.4.1). The code for glacial modeling is available at https://csdms.colorado.edu/wiki/Model:Gc2d. PastElevationChange v1.0, which includes ice cover, palaeo-temperature, digital elevation models of lake catchment, 2006-2300 temperature with RCP4.5 to reproduce the habitat simulation within the lake catchment, have been uploaded to Zenodo (http://doi.org/10.5281/zenodo.4562675). Habitat simulation is implemented in R (version 3.4.3) and QGIS (version 2.16). The statistical analyses are implemented in R (version > 3.4.3) using vegan, psych, codyn, modEvA, and caret package and a re-sampling function for sedaDNA data that archived on Zenodo with identifier doi: http://doi.org/10.5281/zenodo.4562708. The R code for statistical analyses and visualization are available in this published article (Supplementary Code files).

For manuscripts utilizing custom algorithms or software that are central to the research but not yet described in published literature, software must be made available to editors/reviewers. We strongly encourage code deposition in a community repository (e.g. GitHub). See the Nature Research guidelines for submitting code & software for further information.

Data

Policy information about availability of data

All manuscripts must include a data availability statement. This statement should provide the following information, where applicable:

- Accession codes, unique identifiers, or web links for publicly available datasets
- A list of figures that have associated raw data
- A description of any restrictions on data availability

The raw NGS sequencing data that support the findings of this study have been deposited in NCBI Sequence Read Archive (SRA) with the accession code

SRR13957608 (https://www.ncbi.nlm.nih.gov/sra/?term=SRR13957608) and in BioProject PRJNA596631 (https://www.ncbi.nlm.nih.gov/bioproject/PRJNA596631). It also has been archived in addition to the tag-to-sample matrix and taxonomic reference database that support the NGS sequencing data analysis in Dryad Digital Repository with the identifier doi: https://doi.org/10.5061/dryad.vdncjsxth. The filtered sedaDNA datasets analysed during this study are provided in Supplementary Data 1 and Data 2. The data used for statistical analyses are available in Supplementary Data 3. The AMS-dating results and calibrated ages of lake-sediment core can be found at https://doi.org/10.1007/s00334-009-0219-5. The pollen data have been published at https://doi.org/10.1016/j.yqres.2009.12.003 and https://doi.org/10.1016/j.palaeo.2009.12.001. The gridded precipitation data sets are open access and can be downloaded from HAR (https://www.klima.tu-berlin.de/index.php?show=daten_har2&lan=en) and GPCC (https://www.dwd.de/EN/ourservices/gpcc/gpcc.html). The source data underlying Figures. 2a-f and Supplementary Figures 1, 3, 5, and 7 are provided with this paper.

Field-specific reporting

Please select the one below that is the best fit for your research. If you are not sure, read the appropriate sections before making your selection.

☐ Life sciences ☐ Behavioural & social sciences ☒ Ecological, evolutionary & environmental sciences

For a reference copy of the document with all sections, see nature.com/documents/nr-reporting-summary-flat.pdf

Ecological, evolutionary & environmental sciences study design

All studies must disclose on these points even when the disclosure is negative.

Study description	In this study, we reconstruct the total plant taxa richness and taxa richness within alpine plant families) in the catchment area of Lake Naleng (Hengduan Mountains, south-eastern margin of the Tibetan Plateau; Fig. 1a) over the past ~18,000 years. We apply a sedaDNA metabarcoding approach with general plant primers to 72 horizons from a lake sediment core. Observed plant taxa richness changes were numerically related to temperature changes, habitable area, forest shifts and human impact to identify the main driver of changes. Accordingly, we investigate whether total plant taxa richness increases with forest invasion into alpine habitats depicting the modern elevational plant taxa richness pattern. More importantly, we predict plant-richness changes under future climate change by applying the inferred long-term diversity-environmental relationships and provide suggestions for a future plant diversity conservation planning. To support this study, each sediment sample has one DNA isolation, which was replicated until obtaining two positive PCR replicates and associated controls were negative. And, we simulated the glacial activity and habitat area over the past ~18,000 years and the alpine habitat area in 2050-2300 C.E..
Research sample	In total 72 samples were sub-sampled roughly every 250 year from a 17.81 m sediment core. This sediment core is taken from a glacially-formed lake (named Lake Naleng, 31.10° N, 99.75° E, 4200 m a.s.l.) that is situated in south-east Tibetan Plateau, a biodiversity hotspot of the world. The age-depth model for this core had been well-described in a previous study (Kramer et al., 2010, doi: 10.1007/s00334-009-0219-5). With the well-built age-depth model we can track the long-term effects of environment conditions on dynamics of plant diversity using plant DNA metabarcoding approach. This is helpful for predicting future biodiversity and providing valuable strategies for biodiversity conservation.
Sampling strategy	In October 2016, a total of 72 frozen sediment samples at 20- to 40-cm intervals were subsampled in the climate chamber at a temperature of -10°C at the GFZ German Research Centre for Geosciences Potsdam. About 1 mm of the exposed sediment of each sample was removed with a small one-off clean blade and only the inner part of sediment used for ancient DNA isolation. Each blade was used for one side of sediment. The ~ 250-year resolution is sufficient to capture the biodiversity change on the multiple millennium scales.
Data collection	The sub-sampling of lake-sediment samples and DNA isolation from lake-sediment samples, PCR amplification, purification, concentration measurement and pooling were performed by S.L. under the guidance of K.S. The relevant kits and instruments are well described in methods. NGS sequencing was sequenced using Illumina HiSeq 2500 sequencing platform and performed by Fasteris SA which is a Swiss biotechnology company. The (palaeo)environmental datasets, which include temperature, precipitation, Mg/Ca ratio, pollen, current treeline locations, and vegetation information are obtained based on published articles and/or open access database. The detailed information can be found in methods. S.L., S.K.,D.K, and R.H.R contributed to data collection.
Timing and spatial scale	The lake-sediment samples for this study were re-subsampled from the subsampled lake-sediments in October 2016. The DNA isolation, amplification, purification, concentration measurement, pooling were carried out in ancient DNA dedicated facilities at Alfred Wegener Institute, Helmholtz Centre for Polar and Marine Research from November 2016 to March 2017.
Data exclusions	One sample (ID: ESL024) was excluded before statistical analyses because no Plant DNA was detected.
Reproducibility	Each DNA-isolation sample was amplified until obtaining two positive PCR replicates for each lake-sediment sample when the associated controls were negative. After sequencing and data filtering, 6 PCRs were no terrestrial seed plant DNA. Thus, we first test if the potential methodological biases have the impact on the sedaDNA-based plant taxa richness. Then, we test if the plant taxa richness and composition are stable even only one PCR replicate per sample was used. We found that our observed temporal richness pattern and vegetation composition are not sensitive to the dataset used (see Supplementary Table 1 and Supplementary Table 2). Thus, we are confident that the sedaDNA data in our study is a powerful tool for tracking the long-term relative changes of taxa richness.
Randomization	Only in statistic analyses the samples were group by stratigraphically constrained cluster analysis using the “chclust(method = “CONISS”)” in “vegan” based on the relative read abundance.
Blinding	The blinding test is not relevant to this study. We focus on sedimentary ancient DNA from lake and its relationship with palaeoenvironmental changes.

Did the study involve field work? [x] Yes [ ] No

Field work, collection and transport

Field conditions	The fieldwork was conducted in February 2004 to collect the lake-sediment core. The current climatic conditions do not affect the core quality as well as the results of this study.
Location	Lake Naleng (31.10°N 99.75°E, 4,200 m a.s.l.) is located on southeastern of the Tibetan Plateau in Sichuan Province. Max. water depth is 36.7 m.
Access and import/export	The project of sediment collection was between Lanzhou University and Freie Universität Berlin and all rules were followed at that time i.e. no specific permissions were necessary.
Disturbance	No disturbance.

Reporting for specific materials, systems and methods

We require information from authors about some types of materials, experimental systems and methods used in many studies. Here, indicate whether each material, system or method listed is relevant to your study. If you are not sure if a list item applies to your research, read the appropriate section before selecting a response.

Materials & experimental systems

n/a	Involved in the study	
[x]	[ ]	Antibodies
[x]	[ ]	Eukaryotic cell lines
[ ]	[x]	Palaeontology
[x]	[ ]	Animals and other organisms
[x]	[ ]	Human research participants
[x]	[ ]	Clinical data

Methods

n/a	Involved in the study	
[x]	[ ]	ChIP-seq
[x]	[ ]	Flow cytometry
[x]	[ ]	MRI-based neuroimaging

Palaeontology

Specimen provenance	The sediment core is of China provenance. It was collected and transported joint by Lanzhou University and Freie Universität Berlin in 2004. It belongs to project Mi 730/1-1,2 (to S.M.). Not specific documents for permission.
Specimen deposition	The lake-sediment samples are deposited in Alfred-Wegener-Institut für Polar- und Meeresforschung (AWI), Potsdam under project no. 04NC.
Dating methods	There are no new dates collected for this study.

[ ] Tick this box to confirm that the raw and calibrated dates are available in the paper or in Supplementary Information.
